# Production, Quality Control and Biological Evaluation of ^166^Ho-PDTMP as a Possible Bone Palliation Agent

**Published:** 2013-05

**Authors:** Samaneh Zolghadri, Amir Reza Jalilian, Zohreh Naseri, Hassan Yousefnia, Ali Bahrami-Samani, Mohammad Ghannadi-Maragheh, Hossein Afarideh

**Affiliations:** 1 Radiopharmaceutical Research & Development Laboratory (RRDL), Nuclear Science and Technology Research Institute (NSTRI), Tehran, Iran; 2 Faculty of Energy Engineering and Physics, Amirkabir University of Technology, Tehran, Iran

**Keywords:** Biodistribution, ^166^Ho, PDTMP, Radiopharmaceutical, Therapy

## Abstract

***Objective(s):*** In this study, ^166^Ho-1,2-propylene di-amino tetra(methy1enephosphonicAcid) (^166^Ho-PDTMP) complex was prepared as a bone palliation agent.

***Materials and Methods:*** The complex was successfully prepared using an in-house synthesized EDTMP ligand and ^166^HoCl_3_. Ho-166 chloride was obtained by thermal neutron irradiation (1 × 1013 n.cm-2.s-1) of natural Ho(NO_3_)_3_ samples followed by radiolabeling and stability studies. Biodistribution in wild type rats was also peformed.

***Results: ***The complex was prepared with the specific activity of 278 GBq/mg and high radiochemical purity (>99%, checked by ITLC). ^166^Ho-PDTMP complex was stabilized in the final preparation and in the presence of human serum (>90%) up to 72 hr. The biodistribution of ^166^Ho-PDTMP in wild-type rats demonstrated significant bone uptake was up to 48 hr compared to ^166^HoCl_3_.

***Conclusion:*** The produced ^166^Ho-PDTMP properties suggest a possible new bone palliative therapeutic to overcome the metastatic bone pains.

## Introduction

Bone metastases are common in the progression of various tumors such as prostate, breast, and lung carcinoma and they often entail an occurrence of progressive pain (1). Bone metastases occur in many patients with solid malignant tumors (2). Approximately 50% of patients with breast carcinoma and 80% of patients with prostate carcinoma develop metastatic bone disease and nearly half of them experience bone pain (3). In these patients who have progressive disease despite treatment, a systemic bone-avid radiopharmaceutical for treatment of widespread bone metastases has potential benefit (4). Radionuclide therapy using ^32^P, ^89^Sr, ^90^Y, ^153^Sm and ^186^Re has been proposed as an alternative modality for management of bone pain (5). 

Various therapeutic bone-seeking agents have been reported and used in human studies including ^153^Sm-EDTMP (Lexidronam) (6), ^177^Lu-EDTMP (7) and ^166^Ho-DOTMP (8), among those ^153^Sm-EDTMP is the most widely used compound in the world. We have recently reported the production and human application of this compound in the country (9).

Many beta-emitters such as Sm-153, Lu-177 and Ho-166 can be produced in reasonable amounts using (n, gamma) reactions. Holmium-166 (E_β_^-^ max = 1.84 MeV, T_1/2_ = 26.8 hr) is an interesting radionuclides for targeted therapy modalities. Although it is not available in highly specific activities, but the uni-elemental abundance makes it an accessible and inexpensive radionuclide which its obtained specific activity is enough for radiolabeling of small molecules at radiopharmaceutical grades.

**Figure 1:**
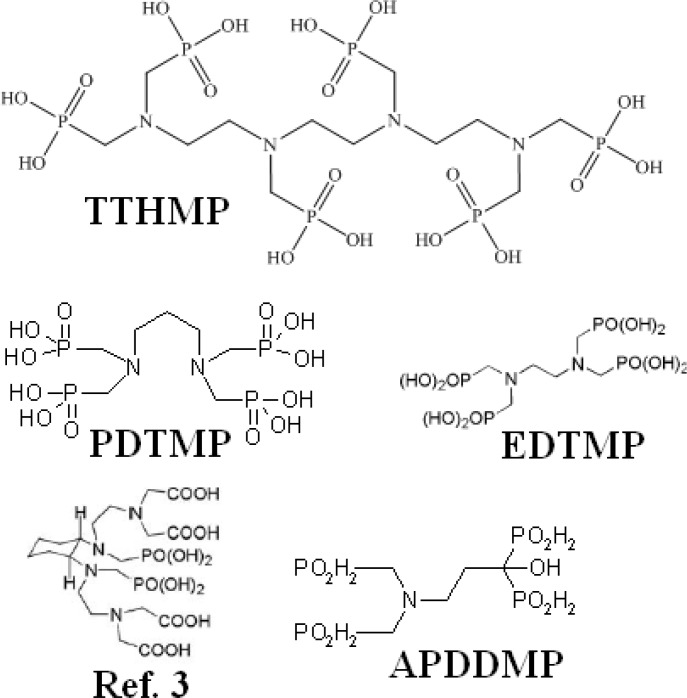
Structures for some phosphonate ligands used in lanthanide labeling

However, the search for the development of new ligands with higher stability, better pharmacokinetics and lower unwanted tissue uptakes (liver and GI) is still ongoing. Various complexes including new cyclic mixed phosphonate/carbonate ligands (10), alkyl phosphonates (PDTMP) (11) and hydroxyl-containing phosphonates (APDDMP) (12) have been developed and evaluated while none of these lanthanides-complexes demonstrated better performance compared to Lexidroinam (Figure 1).

In continuation of developing bone pain palliation agents for the use in the country as well as developing new compounds (9, 13), in this work, we report the preparation, quality control and biodistribution of a new Ho-166 complex of recently synthesized ligand (14), ^166^Ho-1,2-propylene di-amino tetra(methy1enephosphonic acid) (^166^Ho-PDTMP) for ultimate bone pain palliation therapy (Figure 1). 

## Materials and Methods

Production of ^166^Ho was performed at the Tehran Research Reactor (TRR) using ^165^Ho (n, gamma)^166^Ho nuclear reaction. Natural holmium nitrate with purity of >99.99% was obtained from ISOTEC Inc. Whatman No. 1 was obtained from Whatman (Maidstone, UK). Radio-chromatography was performed by using a Bioscan AR-2000 radio TLC scanner instrument (Bioscan, Paris, France). A high purity germanium (HPGe) detector coupled with a Canberra™ (model GC1020-7500SL) multichannel analyzer and a dose calibrator ISOMED 1010 (Dresden, Germany) were used for counting distributed activity in rat organs. All other chemical reagents were purchased from Merck (Darmstadt, Germany). Calculations were based on the 80.6 keV peak for ^166^Ho. Animal studies were performed in accordance with the United Kingdom Biological Council's Guidelines on the Use of Living Animals in Scientific Investigations, 2nd edn. Male healthy rats were purchased from Pasteur Institute, Tehran, Iran. The approval of NSTRI Ethical Committee was obtained for conducting this research. The wild-type rats (NMRI) were purchased from Pasteur Institute of Iran, Karaj, all weighing 180-200 g and were acclimatized at proper rodent diet and 12 hr/12 hr day/night ligh/darkness. 

***Production and quality control of*** ^166^ ***HoCl***_3_ ***solution***

Holmium-166 was produced by neutron irradiation of 100 µg of natural ^165^Ho(NO_3_)_3_ (^165^Ho, 99.99% from ISOTEC Inc) according to reported procedures (15) at the Tehran Research Reactor at a thermal neutron flux of 4×10^13^ n.cm^-2^.s^-1^. Specific activity of the produced ^166^Ho was 5GBq/mg after 20 hr of irradiation. The irradiated target was dissolved in 200 µl of 1.0 M HCl, to prepare ^166^HoCl_3_ and diluted to the appropriate volume with ultra pure water, to produce a stock solution. The mixture was filtered through a 0.22 µm filter (Millipore, Millex GV) and sent for use in the radiolabeling step. The radionuclidic purity of the solution was tested for the presence of other radionuclides using beta spectroscopy as well as HPGe spectroscopy for detection of various interfering beta and gamma emitting radionuclides. The radiochemical purity of the ^166^HoCl_3_ was checked using two solvent systems for instant thin layer chromatography (ITLC)(A: 10mM DTPA pH.4 and B: ammonium acetate 10%:methanol ]1:1[).

*Synthesis of 1,2-propylene di-amino tetra(methy1enephosphonicAcid) (PDTMP)*

The experimental procedure for the synthesis of PDTMP ligand was dependant on other bis-phosphonates as reported (16). Briefly, a quantity of 0.48 g (0.125 mmoles) of 1,2-proylene diamine was dissolved in 0.75 ml of concentrated HC1 and a concentrated aqueous solution of 1.62 g (0.5 mmoles) of phosphorous acid. The resulting solution was heated to reflux temperature and 3.2 ml of 37% aqueous formaldehyde solution (1 mml) was added dropwise in the course of 1 hr to the refluxing solution and refluxing continued for another 2 hr. The result of reaction is an ethanol precipitated of a slightly yellow product from the concentrated reaction solution [m.p. 70-72°C, ^1^H-NMR (D_2_O, δ ppm): 3.02-3.25(m, 12 H, >N-CH_2_CH_2_-N<), 3.37-3.47(m, 12 H, -NCH_2_-PO_3_H_2_)].

***Radiolabeling of PDTMP with*** ^166^ ***HoCl***_3_

A stock solution of PDTMP was prepared by dissolution in 1 N NaOH and diluted to the appropriate volume with ultra pure water by dissolving 250 mg of PDTMP in 1.5 ml NaOH (2N) and 3.5 ml distilled H_2_O, pH. 12. Then 0.3 ml of this solution was added to 200 µl ^166^HoCl_3_ (5.7 mCi) (S.A. 345 mci/mg) and pH adjusted to 7 using phosphate buffer. The reaction mixtures were incubated with stirring at room temperature for 1 hr. Various parameters such as ligand concentration, pH of the reaction mixture, incubation time, reaction temperature were optimized to achieve maximum complexation yield. Sterility, apyrogenicity and toxicity were ascertained by routine methods. The radiolabeling yield of the ligand was determined with paper chromatography using Whatman No. 2 paper by sampling 5µl of the reaction mixture on the paper strip followed by developing in NH_4_OH:MeOH:H_2_O (2:20:40) mixture.

***Stability studies***

The stability of the complex stored at room temperature (22°C ambient), fridge (4°C) and presence of freshly-prepared human serum (at 37°C) was studied at different time intervals by determining the radiochemical purity of the complex by paper chromatography in NH_4_OH:MeOH:H_2_O (2:20:40) system.

***Biodistribution of*** ^166^ ***Ho cation and*** ^166^ ***Ho-PDTMP*** ***in wild-type rats***

To determine its biodistribution, ^166^Ho-PDTMP was administered to normal rats. For comparison, free Ho^3+^ cation buffer solution was also administered. Briefly, 200 μl of final ^166^Ho-PDTMP solution with 0.7 mCi radioactivity was injected intravenously to rats through their tail vein. The animals were sacrificed at the exact time intervals (2, 4, 24 and 48 hr) and specific activity of different organs was calculated as percentage of injected dose per g using HPGe detector (%ID/g).

## Results

***Ligand synthesis***

**Figure 2:**
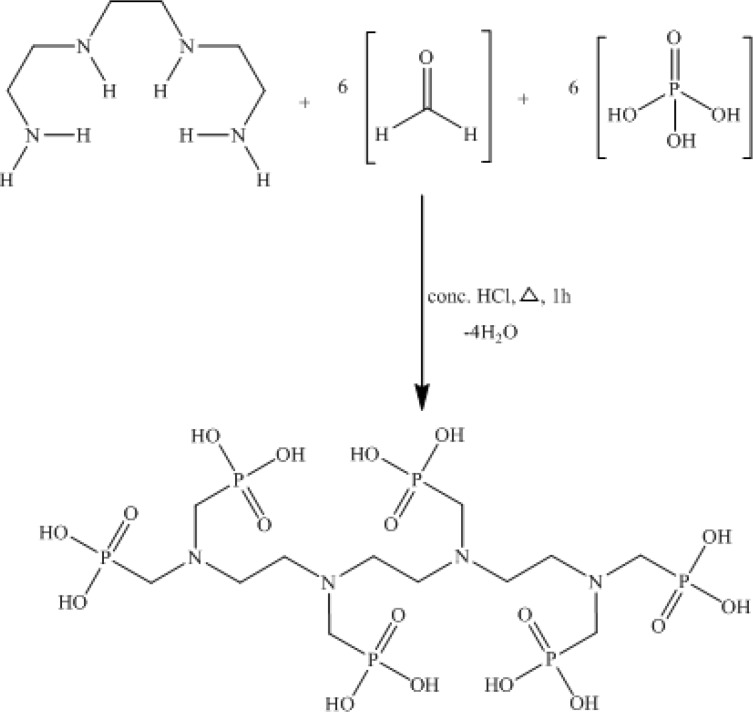
Synthetic scheme of PDTMP

PDTMP ligand was synthesized and the structure was determined using H NMR, C NMR, P NMR and IR methods which was equivalent to other similar authentic samples of bis-phosphonates such as EDTMP and HEDP used in radiopharmacy, according to the conventional method (Figure 2).

***Production and quality control of*** ^166^ **Ho**

**Figure 3:**
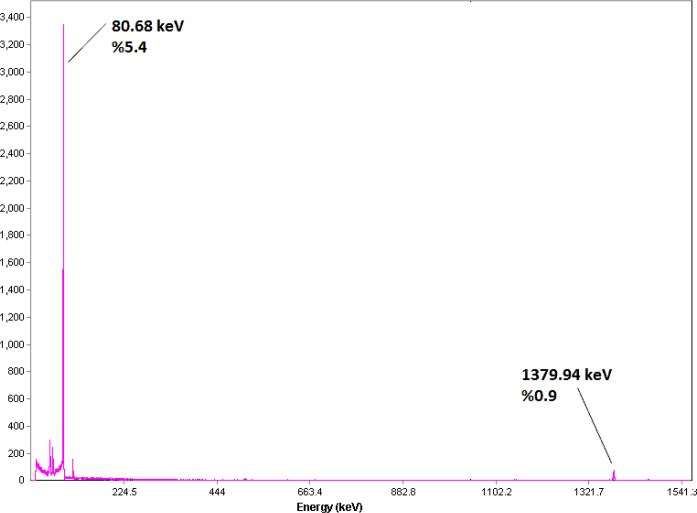
Gamma spectrum for ^166^HoCl_3 _solution used in the radiolabeling

The radionuclide was prepared in a research reactor according to regular methods with a range of specific activity between 3 to 5MBq/mg for radiolabeling use, after counting the samples on an HPGe detector for 5 min and two major photons (5.4% of 80.68 keV and 0.9% of 1379.94 keV) were observed (Figure 3). The radioisotope was dissolved in acidic media as a starting sample and was further diluted and evaporated for obtaining the desired pH and volume followed by sterile filtering.

**Figure 4:**
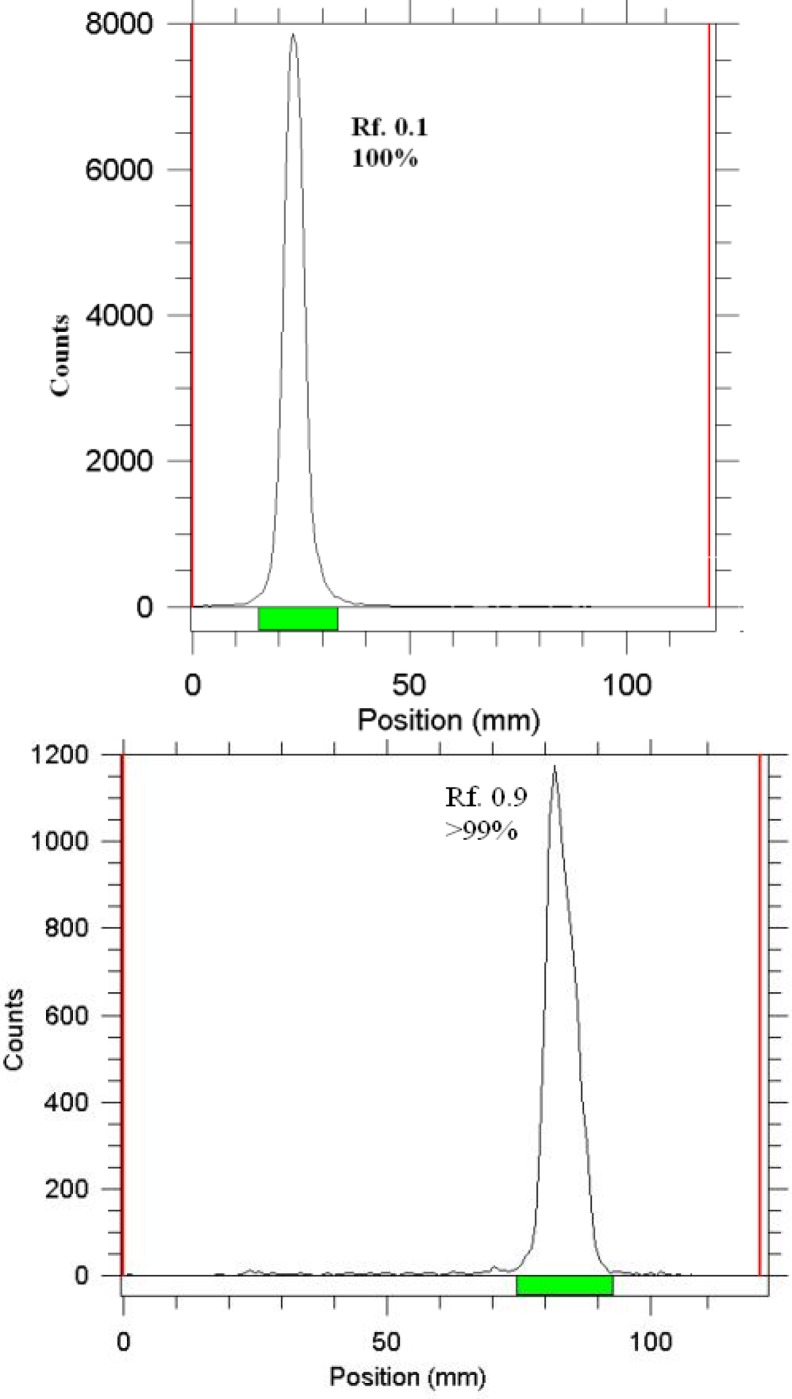
ITLC chromatograms of ^166^HoCl_3_ (above) and ^166^Ho-PDTMP solution (below) using Whatman 1 MM eluted with NH_4_OH: MeOH: H_2_O (0.2:2:4)

The radiochemical purity of the ^166^Ho solution was checked in two solvents. In 10 mmol.L^-1^ DTPA aq. solution (solvent 1), and 10% ammonium acetate:methanol mixture (1:1) (solvent 2) showing the free cation, (Figure 4). The differences of impurity peaks in the two chromatograms could be related to the presence of colloidal impurity (2%). Also about 2% of the activity can be attributed to other ionic impurities. 

***Labeling optimization studies***

In order to obtain maximum complexation yields, several experiments were carried out by varying different reaction parameters such as ligand concentration, pH, reaction time and temperature. Ligand concentration was varied between a wide range starting from 22 to 110 µM/ml for PDTMP. It was observed that at room temperature 99% complexation was achieved with 15 mg/ml of PDTMP. The best ITLC mobile phase was considered Whatman 2 MM paper using NH_4_OH: MeOH: H_2_O (0.2:2:4) as shown in Figure 4.

**Figure 5:**
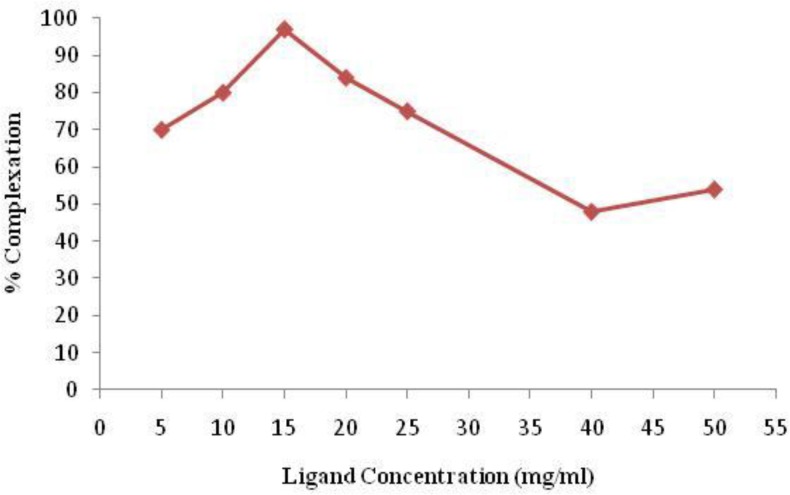
Effect of ligand concentration on complexation yield of ^166^Ho-PDTMP

**Figure 6.**
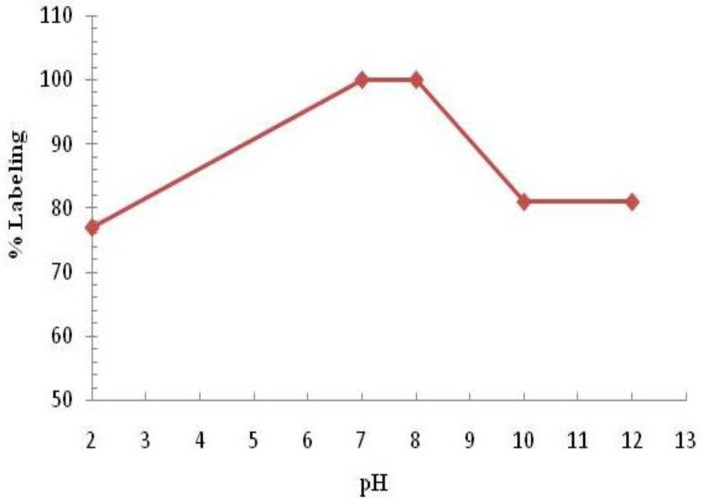
Effect of variation of pH on complexation yield of ^166^Ho-PDTMP at room temperature

Variation of complexation yields with respect to PDTMP concentration is shown in Figure 5. The effect of variation of pH on complexation yield at room temperature was also studied by varying the pH of the reaction mixture from 2 to 12 using 1M HCl or 2M NaOH solution. Maximum yield of 100% was observed at pH 7-8 for complex. The effect of pH on the complexation yield for ^166^Ho-PDTMP complex is shown in Figure 6.

The effect of reaction temperature on complexation yield was not studied for this complex, as sufficiently high complexation yield was achieved at room temperature. The reaction mixture was incubated at room temperature for different time periods and 60 min incubation was found to be adequate to yield maximum complexation.

***Stability***

The stability of the ^166^Ho-PDTMP complex prepared under optimized reaction conditions was studied and observed that the complex showed excellent stability even when stored at room temperature. The complex remained stable to the extent of 96% up to 72 hr, whereas stability of this compound was shown 90% for 72 hr in refrigerator. Usually the higher temperatures accelerate the complex dissociation, however in this case the lower temperature demonstrates the dissociation, that might be explained by the fact that sometimes at this temperature the complex is bond to the borosilica glass vials while the free cation still retains in the mixture and ITLC test would demonstrate higher free cation content leading to lower radiochemical purity and consequently the lower stability (Table 1). demonstrates the assay data of the stability among various temperatures.

***Biodistribution of*** ^166^ ***Ho*** ***cation and ***^166^***Ho****-PDTMP ****in wild-type rats***

**Figure 7:**
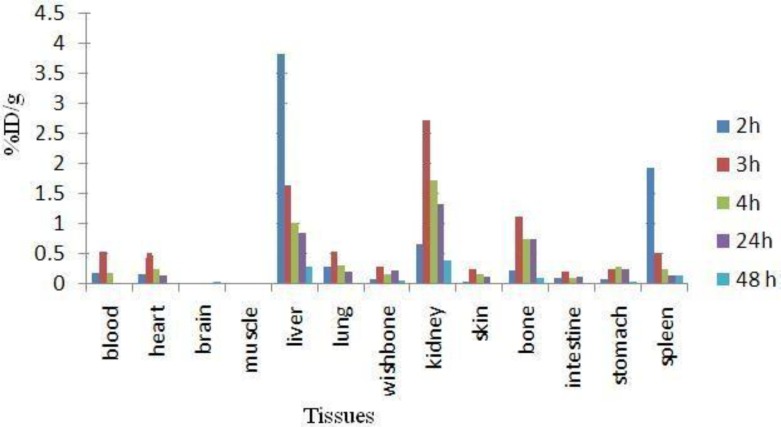
Percentage of injected dose per g (ID/g %) of ^166^HoCl_3_ in rat tissues at 2, 3, 4, 24 and 48 hr post injection

The animals were sacrificed by CO_2_ asphyxiation at selected times after injection (2, 4, 24 and 48 hr). Dissection began by drawing blood from the aorta followed by removing the heart, spleen, muscle, bone, kidneys, liver, intestine, stomach, lungs and skin samples. The tissue uptakes were calculated as the percent of area under the curve of the related photo peak per g of tissue (% ID/g) (Figure 7). The liver uptake of the cation is comparable with many other radio-lanthanides mimicking calcium cation accumulation; about %3 of the activity accumulates in the liver after 48 hr. 

**Table 1:** Stability (%) of the complex at various temperatures

Temp./time	2 hr	4 hr	24 hr	48 hr	72 hr
25C	99	99	98	98	96
4C	99	99	98	95	90

**Figure 8:**
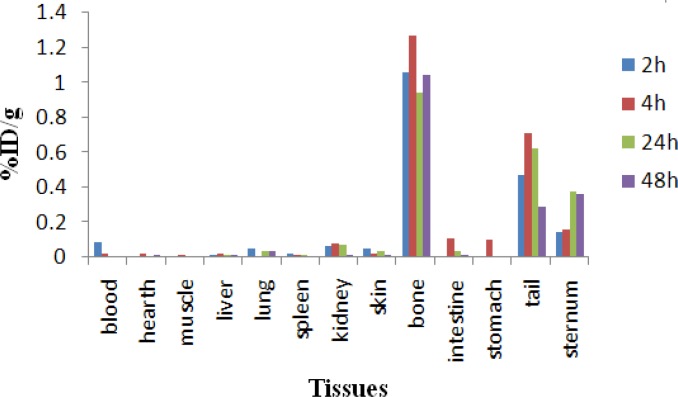
Biodistribution of ^166^Ho-PDTMP in different organs of wild-type rats

For ^166^Ho^3+^ cation, the radioactivity was mainly located in the liver, kidney and bone. The free cation is soluble in water and it can be excreted *via* the urinary tract. Since the metallic ^166^Ho is transferred in plasma into a protein-bond form, the major final accumulation was shown to be in the liver. 

The distribution of injected dose in rat organs up to 48 hr after injection of ^166^Ho-PDTMP (200 µCi/150 ul) solution was determined. Based on these results, it was concluded that the major portion of injected activity of ^166^Ho-PDTMP was extracted from blood circulation into bones (Figure 8).

As shown in Figure 8. The major radioactivity is accumulated in bones as expected for bone-avid radiopharmaceuticals, also due to the presence of anionic properties of the complex and relatively small size of the molecules, the complex is also excreted through the kidneys. Due to liver uptake a significant GI uptake is observed. 

**Figure 9:**
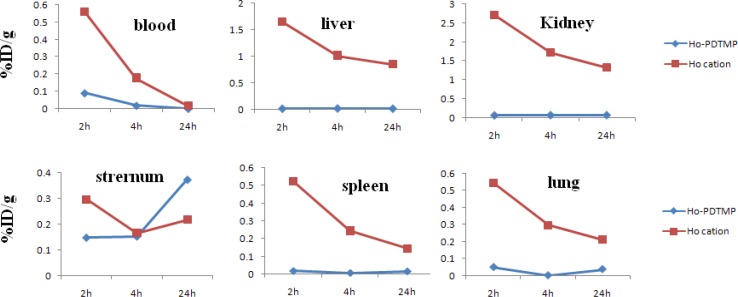
Comparative blood, liver, kidney, sternum, spleen and lung activities (%ID/g) for ^166^Ho-PDTMP and ^166^HoCl_3_ in wild-type rats from 2-24 hr post injection

## Discussion

The radiochemical purity in 10 mmol.L^-1^ DTPA aq. solution (solvent 1), free Ho^3+^ cation is complexed to more lipophilic HoDTPA form and migrates to higher R_f_. Small radioactive fraction remnants at the origin could be related to other Ho ionic species, not forming HoDTPA complex, such as HoCl_4_^-^, etc. and/or colloids. On the other hand, 10% ammonium acetate:methanol mixture (1:1) (solvent 2) was also used for the determination of radiochemical purity. The fast eluting species was possibly Ho^3+^ and other ionic forms of Ho-166 such as HoCl_4_^-^ , remained at the origin (R_f_.0) as well as colloids (Figure 4). The differences of impurity peaks in the two chromatograms could be related to the presence of colloidal impurity (2%). Also about 2% of activity can be attributed to other ionic impurities.

For better comparison of the ^166^Ho-PDTMP and ^66^HoCl_3_ species behavior, Fig 9. demonstrates the tissue accumulation comparison for ^166^Ho-PDTMP and ^166^HoCl_3_.

**Blood:** Both compounds are washed out from the circulation after 48 hr, although the blood wash-out mechanisms are different. 

**Bone**: ^166^Ho-PDTMP is rapidly taken up in bones in 2 hr after administration and retains almost constantly up to 24 hr. Instead, ^166^Ho cation uptake slowly increases but never exceeds %1.

**Kidney:** As mentioned earlier, ^166^Ho-PDTMP is rapidly taken up in bones and the trapping continued in a way that almost no blood circulation activity as well as kidney excretion can be observed. Instead, as a water soluble cation most of free Ho-166 activity is washed out through kidney in 48 hr.

**Liver:** A major difference in liver uptake is observed for two species. ^166^Ho-PDTMP has almost no liver accumulation, which is a major advantage as a therapeutic radiopharmaceutical due to the possibility of increasing the maximum administered dose compared to other bone seeking therapeutic radiopharmaceuticals such as ^177^Lu-EDTMP and ^153^Sm-EDTMP. While Ho^3+^ cation, being transferred by serum metalloproteins, accumulates in liver and is excreted through hepatobilliary excretion route, leading to the reduction in liver accumulation.

**Spleen:** ^166^Ho-EDTMP almost is not accumulated in spleen which can be again a major advantage as a therapeutic radiopharmaceutical due to the possibility of increasing the maximum administered dose, while Ho-166 cation is present in spleen 2 hr post injection while slowly is washed out in 48 hr.

## Conclusion

For ^166^Ho-PDTMP the radiochemical purity was higher than 99% and the labeling and quality control took one hour. The radiolabled Lu complex was prepared in high radiochemical purity (>99%, ITLC) and specific activity of 278 GBq/mmol and demonstrated significant stability at 4, 25 and 37C (in presence of human serum). The final preparation was administered to wild-type rats and biodistribution of the radiopharmaceutical was checked 4 hr-7day later showing major accumulation of the drug in the bone tissues. ^166^Ho-PDTMP can be a probable candidate for bone pain palliation therapy in skeletal metastases, although further biological studies in other mammals is still needed.

## Authors’ Statements

The authors declare no conflict of interest. The results described in this paper were part of student thesis. 

## References

[1] Serafini AN (2001). Therapy of metastatic bone pain. J Nucl Med.

[2] Bayouth JE, Macey DJ, Kasi LP, Fossella FV (1994). Dosimetry and toxicity of Samarium-153-EDTMP administered for bone pain to skeletal metastases. J Nucl Med.

[3] Campa JA, Rayne R (1992). The management of intractable bone pain: a clinician’s perspective. Semin Nucl Med.

[4] Eary JF, Collin C, Stabin M, Vernon C, Petersdorf S, Baker M (1993). Samarium-153-EDTMP biodistribution and dosimetry estimation. J Nucl Med.

[5] Holmes A (1992). 153Sm-EDTMP: a potential therapy for bone cancer. Semin Nucl Med.

[6] Serafini AN, Houston SJ, Resche I, Quick DP, Grund FM, Ell PJ (1998). Palliation of pain associated with metastatic bone cancer using samarium-153 lexidronam: a double-blind pacebo-controlled clinical trial. J Clin Oncol.

[7] Máthé D, Balogh L, Polyák A, Király R, Márián T, Pawlak D (2010). Multispecies animal investigation on biodistribution, pharmacokinetics and toxicity of 177Lu-EDTMP, a potential bone pain palliation agent. Nucl Med Biol.

[8] Breitz HB, Wendt III RE, Stabin MS, Shen S, Erwin WD, Rajendrann JG (2006). 166Ho-DOTMP radiation-absorbed dose estimation for skeletal targeted radiotherapy. J Nucl Med.

[9] Bahrami-Samani A, Ghannadi-Maragheh M, Jalilian AR, Meftahi M, Shirvani-Arani S, Moradkhani S (2009). Production, quality control and biological evaluation of 153Sm-EDTMP in wild-type rodents. Iran J Nucl Med.

[10] Ouadi A, Loussouarn A, Morandeau L, Remaud P, Faivre-Chauvet A, Webb J (2004). Influence of trans-1,2-diaminocyclohexane structure and mixed carboxylic/phosphonic group combinations on samarium-153 chelation capacity and stability. Eur J Med Chem.

[11] Majali MA, Mathakar AR, Shimpi HH, Banerjeea S, Samuel G (2000). Studies on the preparation and stability of samarium-153 propylene diamine tetramethylene phosphonate (PDTMP) complex as a bone seeker. Appl Radiat Iso.

[12] Zeevaart JR, Jarvis NV, Werner KA, Jackson GE (2001). Metal-ion speciation in blood plasma incorporating the tetraphosphonate, N,N-dimethylenephosphonate-1-hydroxy-4-aminopropilydenediphosphonate (APDDMP), in therapeutic radiopharmaceuticals. J Inorg Biochem.

[13] Bahrami-Samani A, Bagheri R, Jalilian AR, Shirvani-Arani S, Ghannadi-Maragheh M, Shamsaee M (2010). Production, quality control and pharmacokinetic studies of Ho-EDTMP for therapeutic applications. Sci Pharm.

[14] Chakraborthy S, Das T, Unni PR, Sarma HD, Samuel G, Banerjee S (2002). 177Lu-labelled polyaminophosphonates as potential agents for bone pain palliation. Nucl Med Commun.

[15] ( 2003). Manual for reactor produced radioisotopes.

[16] Moedritzer K, Irani RR (1996). Direct synthesis of α- aminomethyl phosphonic acid: mannich type reactions with o- phosphorus acid. J Org Chem.

